# Cirrhotic Cardiomyopathy: The Interplay Between Liver and Heart

**DOI:** 10.7759/cureus.27969

**Published:** 2022-08-13

**Authors:** Revanth Kalluru, Sai Gadde, Rahul Chikatimalla, Thejaswi Dasaradhan, Jancy Koneti, Swathi priya Cherukuri

**Affiliations:** 1 Research, Kamineni Institute of Medical Sciences, Narketpally, IND; 2 Research, Narayana Medical College, Nellore, IND

**Keywords:** myocardial strain imaging, cirrhotic cardiomyopathy consortium criteria, diastolic dysfunction, cardio hepatorenal syndrome, systolic dysfunction, cardiac cirrhosis, cirrhotic cardiomyopathy

## Abstract

Cardiac vascular dysfunction was described years ago in alcohol-associated liver cirrhosis and recently became known as cirrhotic cardiomyopathy (CCM) in 2005. Cirrhotic cardiomyopathy is a specific cardiac dysfunction estimated to be prevalent in half of the liver cirrhosis patient population; it comprises a triad of impaired myocardial contractile responses to stress (systolic dysfunction), inadequate ventricular relaxation, and electrophysiological abnormalities. This review describes the various pathophysiological mechanisms connecting liver cirrhosis to the alterations seen in CCM and briefly mentions the role of the cardiovascular system in connecting the pathophysiology of hepatorenal syndrome (HRS). Insertion of the transjugular intrahepatic portosystemic shunt (TIPS) and liver transplantation exacerbates the underlying cardiac dysfunction leading to signs and symptoms of heart failure. This article also focuses on the clinical importance of diagnosing CCM and the limitations existing around traditional diagnostic criteria based on transmitral flow parameters. It highlights newer parameters proposed by the Cirrhotic Cardiomyopathy Consortium to obtain a diagnosis of CCM. Liver transplantation is the only treatment available to cure CCM.

## Introduction and background

The term hepatic cirrhosis was first used by René Laënnec, a French physician referring to its tawny appearance (*kirrhos* in Greek) [1]. Cirrhosis is defined by its characteristic histological changes in regenerative islands of hepatocytes forming nodules and extensive surrounding fibrous septae due to chronic liver injury [2]. Cirrhosis causes 1.03 million deaths per year worldwide and 33,539 deaths per year in the USA [3,4]. It is prevalent worldwide, and the exact number may be difficult to ascertain due to its indolent clinical course. Alcohol abuse and hepatitis C virus are the leading causes of cirrhosis among Caucasians, however, non-alcoholic fatty liver disease is rising in prevalence. Among the African and Asian populations, the hepatitis B virus remains the most common causative organism [5].

The hepatocytes are the primary site of insult and drive the pathophysiology of cirrhosis. Key players in the pathogenesis are activated hepatic stellate cells (HSCs), Kupffer cells, and cytokines like platelet-derived growth factor, transforming growth factor beta (TGF-β), and others. The pathogenesis of cirrhosis involves anatomical and functional alterations [5,6]. Parenchymal extinction lesions with islands of regeneration, fibrogenesis, defenestration, and capillarization of sinusoidal endothelial cells are characteristic features. Functional changes include reduced production of vasodilators and increased production and responsiveness to vasoconstrictors, further contributing to hepatic resistance and a rise in portal venous pressure [5,6].

Cirrhosis may be asymptomatic or brought to clinical attention due to loss of hepatocyte function and increased intrahepatic resistance (portal hypertension and its sequelae). It may progress to multisystem involvement (hepatorenal syndrome (HRS) and hepatopulmonary syndrome), and hepatocellular carcinoma (HCC) [2,7,8]. Asymptomatic cases are discovered incidentally on routine liver function tests (LFTs) and imaging and confirmed on additional evaluation [2]. Non-invasive methods like FibroScan® (Echosens, Paris, France) help determine the extent of fibrosis [2]. Several classifications are used to determine the severity and for predicting the prognosis and outcomes of treatment, like the Child-Pugh Turcotte (CPT) classification and model for end-stage liver disease (MELD) [9,10]. Eliminating causative agents is the first step to modifying the progression of cirrhosis, and certain studies suggested a reversal of cirrhosis [2,11].

Cirrhotic cardiomyopathy (CCM) is a complex cardiac dysfunction among cirrhotics in the absence of prior cardiac pathology and it is independent of the etiology of liver cirrhosis [12,13]. About 50% of liver cirrhosis patients are estimated to have an underlying cardiac dysfunction [14,15]. Current research believes CCM drives multisystem complications following liver cirrhosis like HRS [7]. Further, a treatment explicitly targeting CCM does not exist and is yet to be developed [16]. Insertion of the transjugular intrahepatic portosystemic shunt (TIPS), and liver transplantation can exacerbate the underlying cardiac dysfunction and lead to heart failure. The latter is associated with several cardiac complications [17,18]. An understanding of the underlying pathophysiologic mechanisms, defining parameters, and modern diagnostic modalities are essential. This article aims to discuss pathogenic mechanisms, highlight the current defining and diagnostic criteria and outline the currently available treatment options.

## Review

Origin of the term cirrhotic cardiomyopathy

The liver and heart are well known to be conjointly involved in the disease processes of one another. Heart failure (HF) may lead to acute cardiogenic liver injury (formerly shock liver) and congestive hepatopathy, eventually leading to cirrhosis [19-22]. On the other hand, liver cirrhosis, Wilson's disease, and hemochromatosis lead to cardiac dysfunction without a prior cardiac pathology [23,24]. This review focuses on the cardiovascular abnormalities led by liver cirrhosis. Cirrhosis due to alcohol abuse may take credit for the unfolding of cardiac abnormalities, then called alcoholic heart muscle disease [25]. Alcoholic cirrhosis patients have increased cardiac output (CO) and a prolonged QT interval. These changes were initially presumed to be due to thiamine deficiency and the direct toxic effects of alcohol [26,27]. Following a liver transplant, about 40% to 50 % of patients with cirrhosis developed HF [26]. After that, studies described that exercise had a subnormal response to CO and stroke volume (SV) [28]. These findings pointed to an underlying cardiac abnormality. Several autopsy studies of cirrhotic patients showed structural alteration of the heart chambers, proving that liver cirrhosis is associated with cardiomyopathy in the absence of prior heart disease and is independent of the etiology of liver cirrhosis per se [12,13].

The term CCM was first used in 2005 at the World Conference of Gastroenterology in Montreal, Canada. It is a distinct type of cardiac dysfunction that ensues following a cascade of events driven by liver cirrhosis and not a mere structural change to the heart [16]. It denotes a triad, the blunted contractile response to stress (systolic dysfunction), impaired ventricular relaxation (diastolic dysfunction), and electrophysiological abnormalities in the absence of any known cardiac disease [22,23]. A crucial element to note is that the etiology of liver cirrhosis makes no difference, and cases are also reported in children [12,13,29]. Cirrhotic cardiomyopathy is often asymptomatic for years due to a normal CO at rest and an indolent disease course. It comes to clinical attention during the decompensated stage of cirrhosis with features of diastolic dysfunction [30]. This poses a challenge in determining the exact number of CCM cases. However, it is estimated that about 50% of patients with cirrhosis have underlying CCM [14,15]. Depending on the selection criteria used for a study, the underlying etiology for cirrhosis in such patients can vary. A cross-sectional observational study by Kazanov et al. in 2011 found 73% of patients with cirrhosis to be of alcoholic origin [14]. Another study by Nazar et al. concluded that the etiology of cirrhosis was alcoholic in 45% of the patients and hepatitis C-associated in 40% [15].

Pathophysiologic events

The complex events following liver cirrhosis leading up to CCM are discussed in two events. One is the effect of liver cirrhosis on circulation, and two is on the heart itself. 

Liver Cirrhosis and Circulatory Changes in CCM

Liver cirrhosis is often asymptomatic in its early stages, called compensated cirrhosis. An estimated 5% to 7% of them per year progress to portal hypertension and/or hepatic failure, known as decompensated cirrhosis [31]. Portal hypertension is driven by two crucial changes in the liver, mainly by structural derangements and further by functional components. In the liver, decreased production of vasodilators, mainly nitric oxide (NO), increased production, and increased responsiveness to vasoconstrictors like thromboxane A2 affect the contractility of HSCs and myofibroblasts in sinusoids [5]. In contrast to reduced sinusoidal NO production, there is a compensatory excess vasodilator drive in the peripheral arterial circulation, especially leading to splanchnic vasodilation [32,33]. This drive is thought to be an attempt to oppose portal hypertension. Vasodilators NO, carbon monoxide (CO), and endocannabinoids escape hepatic degradation due to liver metabolic dysfunction and via portosystemic shunting. They are further compensatively released and exert their effects on peripheral circulation [32-34]. The arterial vasodilation hypothesis states that peripheral arterial dilation, especially splanchnic, is believed to be the central event in the subsequent development of the hyperdynamic syndrome [35]. These molecules also affect the contractility of the heart, which is discussed later in this article.

**Figure 1:**
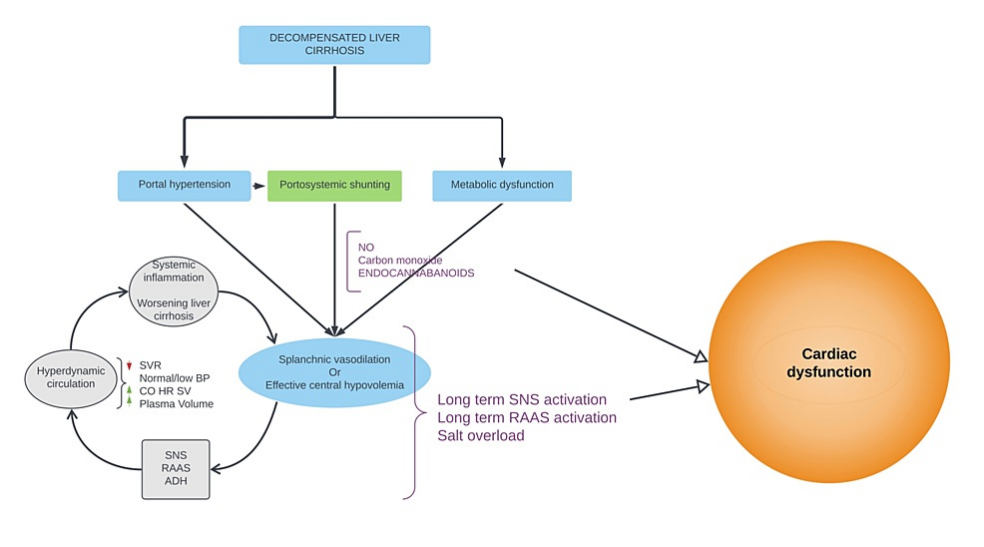
Pathogenic events following liver cirrhosis leading up to CCM CCM: Cirrhotic cardiomyopathy, NO: Nitric oxide, SNS: Sympathetic nervous system, RAAS: Renin-angiotensin-aldosterone system, ADH: Antidiuretic hormone, SVR: Systemic vascular resistance, CO: Cardiac output, HR: Heart rate, SV: Stroke volume, BP: Blood pressure Image credits: Figure created by author Revanth Kalluru

Lowered systemic vascular resistance (SVR) due to progressive arterial dilation, a compensatory increase in CO, SV, heart rate (HR), and a normal/low-normal blood pressure (BP) constitute the hyperdynamic syndrome in cirrhosis [35]. In the compensated stage of cirrhosis, blood pressure is maintained by increasing CO as the drop in SVR is only mild [36]. It is only in the decompensated stage of cirrhosis that a marked decrease in SVR due to vasodilators escaping hepatic degradation is observed (Figure 1) [32-35]. This phenomenon slightly drops the BP, and peripheral blood starts redistributing to the splanchnic circulation. Low effective blood volume leads to a drop in glomerular filtration rate (GFR) and volume receptor activation, and low arterial tension leads to baroreceptor activation in the carotid sinus resulting in activation of potent vasoconstriction systems like the renin-angiotensin-aldosterone system (RAAS), sympathetic nervous system (SNS), and vasopressin system [37]. They attempt to maintain the blood volume by retaining sodium and water and maintain the blood pressure and organ perfusion by increasing CO, HR, SV, and arterial tone (Figure 1) [23]. However, as cirrhosis decompensation progresses, SVR worsens, and blood redistributes more in the splanchnic circulation, worsening central blood volume known as central hypovolemia [38,39]. As a result, a vicious cycle starts where a high CO and plasma volume increase cannot compensate for a low SVR, thereby continuously activating potent vasoconstrictor systems leading to the hyperdynamic syndrome and splanchnic plasma volume increase (Figure 1) [38,39]. Kowalski et al. performed a study in 1953 and described circulatory changes in liver cirrhosis (increase in CO and increase in HR) which were later recognized as a part of the hyperdynamic syndrome [40]. Long-term activation of potent vasoconstrictor systems and molecules escaping hepatic degradation described above conspire together to cause cardiac dysfunction as shown in Figure 1.

Additional factors playing a role in a hyperdynamic syndrome that are worth mentioning are the role of the vasopressin system and systemic inflammation [41-43]. Copeptin (a fragment of the vasopressin precursor released from the posterior pituitary gland) levels increase with the progression of cirrhosis. Also, they increase significantly in those who would develop complications of cirrhosis in the future [41]. Interestingly systemic inflammation has a strong association with the pathogenesis of CCM. Following impaired mucosal defense and increased intestinal permeability, bacteria and pathogen-associated molecular patterns (PAMPs) are believed to migrate from the intestine to extra-intestinal organs. Subsequently, activating the immune system leads to the release of inflammatory cytokines and vasodilators like NO. These mediators worsen the splanchnic vasodilation, further fueling the vicious cycle [42]. The plasma level of lipopolysaccharide-binding protein (LBP), a marker for bacterial endotoxin exposure, is related to the severity/degree of diastolic dysfunction [43].

Liver Cirrhosis and Cardiac Dysfunction in CCM: Systolic Dysfunction

Systolic dysfunction, in general, refers to an emptying problem of the left ventricle (LV), described by a decreased ejection fraction (EF), usually as a result of an impaired myocardial contractile function [44,45]. Systolic dysfunction in CCM, according to the world conference of gastroenterology 2005 criteria, is defined as left ventricular ejection fraction (LVEF) <55% at rest and/or blunted contractile response to stress [46]. At rest, CCM is well tolerated and asymptomatic. The LVEF in CCM is normal or even higher due to the peripheral arterial dilation leading to reduced afterload on the heart. Thus, the LV’s underlying contractile dysfunction is masked in patients at rest [9,35]. Introduction of physiologic stress (in the form of exercise), pharmacologic stress, or liver transplant may reveal the underlying latent systolic dysfunction [28,47-49]. Wong et al. in 2001 performed a study to determine the influence of exercise on the myocardium in 39 cirrhosis, and 12 controls matched by age and sex concluded that an increase in volume load and pressure to the heart during exercise failed to increase the CO and EF simultaneously [28]. Based on a study by Krag et al. on the administration of terlipressin, a reduced CO and EF were noticed following increased afterload on the heart [47]. These results indicate that myocardial contractile dysfunction (systolic dysfunction) was always present in these patients. The hyperdynamic state with normal or high CO masked signs and symptoms of HF. It also infers that the myocardium fails to increase its performance to an increase in demand, also called chronotropic incompetence, partly due to an already baseline high CO and other mechanisms [28,47-49]. Similarly, Sampathkumar et al. reported a post-liver transplant decrease in LVEF from 60% to 20%, suggesting a sudden increase in afterload post-liver transplant due to reversal of hyperdynamic state depressed the cardiac performance [49].

Several hypotheses have been put forward to describe the impaired contractility and broadly are due to altered β adrenergic signaling pathway and elevated cardio depressant molecules reaching the heart (as shown above in Figure 1) [50-53]. The SNS hyperactivity, although it aims to restore cardiovascular function initially, due to repeated long-term activation as a part of the hyperdynamic syndrome contributes to the pathophysiology of CCM. Several studies show a decrease in the number of β adrenergic receptors on the myocardial cell membrane (downregulation) and altering β adrenergic receptor signaling by decreasing cyclic adenosine monophosphate (cAMP) production due to uncoupling of G protein from β adrenergic receptor (desensitization) [50-52]. These changes are believed to be a consequence of constant stimulation by increased catecholamines. Understanding cardiac action potential coupling with mechanical ventricular systole is imperative to understand the role of cardio depressant molecules better. Depolarization of the cardiomyocytes' plasma membrane leads to calcium ion influx due to activation of L-type voltage-gated calcium channels. It drives calcium-induced calcium release from the sarcoplasmic reticulum via the ryanodine receptor (RyR) [54]. Myocardial relaxation follows the normalization of intracellular calcium by sarcoplasmic reticulum reuptake and expulsion out to the cytosol by calcium pumps (adenosine triphosphate dependent) and sodium-calcium exchanger (Na+ calcium ion (Ca2+) exchanger-ion gradient dependent), respectively [54]. Cardio depressant molecules NO, carbon monoxide, and endocannabinoids influence this excitation-contraction coupling and also alter β adrenergic receptor signaling [31,53,55,56].

Nitric oxide, in general, is cardioprotective, enhances perfusion, and inhibits apoptosis of cardiomyocytes. This NO is derived from endothelial nitric oxide synthase located in the pits of plasmalemma called caveolae [26]. In the setting of decompensated cirrhosis, inflammatory cytokines (tumor necrosis factor-alpha (TNF-α) and interleukin-1b) upregulate inducible NO synthase (iNOS), and the NO produced as a result has a cardiotoxic effect [53,57]. Nitric oxide exerts intracellular action via its second messenger cyclic guanosine 3′,5′ monophosphate (cGMP). The cGMP-dependent protein kinases (PKGs) exert negative ionotropic and apoptotic effects on cardiomyocytes via phosphorylation of various targets [58]. The cGMP is believed to 1) inhibit L-type calcium channels, thereby reducing calcium influx; 2) inhibit RyR of the sarcoplasmic reticulum, decreasing calcium-mediated calcium release; 3) promote degradation of cAMP, which impairs β adrenergic signaling [59]. Nitric oxide may also affect the pacemaker by inhibiting calcium influx and altering β-adrenergic stimulation to cause bradycardia [60]. Carbon monoxide is also believed to mediate its cardio depressive effects via cGMP and cGMP-dependent protein kinases (PKGs) [55]. Van Obbergh et al. studied cardiac modification in bile duct ligation (BDL) rats and concluded that NO impaired contractility of the heart, and adding a NOS inhibitor significantly improved contractility [61].

Endogenous cannabinoids are lipids synthesized from the cell membrane phospholipids as and when the cell requires them. They act via a G protein-coupled cannabinoid (CB) receptor [62,63]. The cannabinoid receptor type 1 (CB1) receptors are thought to be expressed in the heart, endothelial cells, brain, and many other sites [62]. Endocannabinoids are believed to exert a negative ionotropic effect on the heart leading to systolic dysfunction [26,64]. A study performed by Yang et al. in 2010 on BDL mice to study the interactions of various molecules on cardiodepression via CB receptors concluded that anandamide (AEA) (an endocannabinoid) synthesis was induced by TNFα (an inflammatory cytokine) nuclear factor kappa B (NFkappaB) iNOS signaling pathway, which leads to depressed contractility [64]. In the same study administering CB1 antagonist and endocannabinoid reuptake inhibitor improved contractility and worsened contractility, respectively [64]. Thus, systemic inflammation triggered release of cytokines may subsequently depress systolic function via endocannabinoid release [16,64]. It is believed that the negative ionotropic effect via the CB1 receptor (a G protein-coupled receptor) is by interfering with β adrenergic signaling (reducing intracellular cAMP) and inhibiting calcium influx (L-type calcium channel inactivation) [26].

Recent studies suggest cardiomyocyte apoptosis plays a role in the pathogenesis of systolic dysfunction [65]. A study performed by Nam et al. in 2014 on BDL rats to study the influence of apoptotic pathways on ventricular contractility concluded that injection of anti-Fas ligand monoclonal antibody improved cardiac contractility only in BDL rats and not in controls suggesting a potential role of apoptosis in systolic dysfunction [65]. The TGF-β production due to SNS hyperactivity is believed to be the main culprit [16]. The TGF-β is a proapoptotic cytokine and promotes cardiomyocyte apoptosis by activating mitogen-activated protein kinases (MAPK) [66]. A persistent elevation of intracellular Ca^2+ ^in cardiomyocytes is associated with cardiomyocyte apoptosis [67]. Following an action potential induced Ca^2^^+^ influx into a cardiomyocyte, the plasma membrane Na^+^/Ca^2+^ exchanger extrudes 15% of calcium influx, and the rest is sequestered into the sarcoplasmic reticulum. Crespo et al. showed that by inhibiting the Na^+^/Ca^2+^ exchanger, the cardiomyocyte could not extrude intracellular Ca^2+ ^completely to a steady state level. Although it enhances contractility initially, a persistently elevated resting intracellular Ca^2+^ led to cardiomyocyte apoptosis [68].

The current understanding of the complications in end-stage liver disease is that systolic dysfunction partly drives the progression to HRS [69]. Hepatorenal syndrome is characterized by a reduction in renal blood flow and GFR in cirrhotic patients and the absence of any evidence for intrinsic kidney disease [69,70]. Based on a normal or even higher CO in hyperdynamic circulation, an underlying cardiac dysfunction in HRS was not questioned initially [26,35,69]. The traditional understanding of HRS is based solely on lowered SVR leading to central hypovolemia and renal vasoconstriction owing to the vasoconstrictor systems [69]. A study performed by Ruiz-del-Arbol et al. in 2005, investigated the cardiac function and hemodynamics in 66 cirrhotic patients with tense ascites before and after HRS development and concluded that mean arterial pressure and CO decreased significantly in the 27 patients who further developed HRS on follow-up compared to those who did not progress to HRS [71]. Plasma renin and plasma norepinephrine were significantly higher, but SVR remained unchanged between those who developed HRS and those who did not (Table 1) [71]. This suggested that in end-stage liver disease, a decline in afterload is not compensated by increasing the CO further and cannot compensate for the central hypovolemia precipitating the HRS [69]. It led to discarding the traditional understanding of HRS, where cardiac and renal systems were believed to be separately involved in liver cirrhosis. It is now hypothesized that underlying cardiac dysfunction drives the HRS's pathogenesis in a liver cirrhosis setting, suggesting the entity cardiorenal syndrome (CRS)/hepatocardiorenal syndrome (Table 1) [69]. Venous congestion due to cardiac dysfunction and plasma volume overload may further lead to increased renal venous pressure impairing renal blood flow [72]. In the study by Ruiz-del-Arbol et al., CO and SV were also lower initially in those who would subsequently develop HRS. This suggested that cardiac dysfunction precedes renal impairment and drives the pathogenesis of HRS [69,71].

**Table 1:** A summary of studies to understand circulatory dysfunction and systolic dysfunction in cirrhosis BDL: Bile duct ligation, TNF-alpha: Tumor necrosis factor alpha, AEA: Cardiac anandamide, CB1 receptor: Cannabinoid receptor, CCM: Cirrhotic cardiomyopathy, MAP: Mean arterial pressure, CO: Cardiac output, HRS: Hepatorenal syndrome, HR: Heart rate, bpm: beats per minute, EF: Ejection fraction, CI: Cardiac index, NOS: Nitric oxide synthase

Reference	Population	Methods	Results	Conclusion
Yang et al. [64]	68 rats ( 37 cirrhotic rats obtained by BDL and 31 age-matched control rats	Plasma TNF-alpha effect on AEA was studied.	Plasma TNF-alpha level and its signaling pathway mediators expression increased leading to increased AEA production. Cardiac contractility was blunted in BDL mice. Administration of CB1 receptor antagonist improved the contractility whereas, cannabinoid reuptake inhibitor worsened it.	Systemic inflammation triggered the release of cytokines led to subsequent depressed systolic function via endocannabinoid release.
Nam et al. (2014) [65]	BDL rats and control sham-operated rats	Determining the influence of intrinsic and extrinsic apoptotic pathways on ventricular contractility using immunohistochemical staining and western blot analysis.	Injection of anti-Fas ligand monoclonal antibody improved cardiac contractility only in BDL rats and not in controls suggesting.	Cardiomyocyte apoptosis plays a potential role in the systolic dysfunction of CCM.
Ruiz-del-Arbol L et al. (2005) [71]	66 patients had liver cirrhosis with tense ascites and a normal serum creatinine level on admission	Hemodynamic state in cirrhotics before and after the development of hepatorenal syndrome (HRS) was analyzed.	Baseline MAP and CO were significantly lower in patients who developed HRS compared to those who have not developed HRS. Plasma renin activity and norepinephrine concentration were higher in patients who developed HRS compared to those who have not developed HRS. Systemic vascular resistance remained unchanged.	In the end-stage liver disease, a decline in afterload is not followed by an increase in the cardiac output thus, unable to compensate for the central hypovolemia leading to HRS.
Wong et al. (2001) [28]	51 patients (39 liver cirrhotics and 12 age and sex-matched controls	Based on radionuclide angiography and graded upright cycle ergometry, cardiac function was assessed before and after exercise.	Peak HR following exercise in controls was 147 bpm and 110 bpm in cirrhotics with ascites Increase in cardiac output following exercise was lower in cirrhotics.	Due to exercise, patients with liver cirrhosis exhibited chronotropic and ionotropic incompetence.
Krag et al. (2010) [47]	24 patients with liver cirrhosis and ascites	MAP, EF, and CO were compared before and after exercise following 2mg terlipressin administration.	CO and EF dropped by 17% and 16%, respectively, after an increase in MAP following terlipressin administration compared to a 1% increase in EF and a 2% fall in C.O in the placebo group.	On stressing the heart in cirrhotics by increasing afterload a blunted contractile response was noticed.
Kowalski et al. (1953) [40]	22 patients with parenchymal liver disease (19 with cirrhosis and without any evidence of prior heart disease )	Dye injection method by Hamilton et al. was used to determine the CO and CI was calculated further.	Mean CI of all the patients was 4.26 +/- 2.73 L. per m2. Seven among 22 had CI values above the normal range. None of them had values below the normal range.	An elevated baseline CO and heart rate were found in patients with liver disease and later came to be known as a part of the hyperdynamic syndrome.
van Obbergh et al. (1996) [61]	8 (BDL) rats and 8 control rats	Systolic ventricular pressure before and after NOS inhibitor infusion were compared.	Cirrhotic rats had a lower systolic pressure (58.9 +/- 7.4 mmHg) compared to controls (80 +/- 4.4 mmHg). Following NOS inhibitor infusion no systolic pressure increased in cirrhotic rats (to 68.5 +/- mmHg) compared to no significant change in controls.	Nitric oxide is believed to impair the contractility of the heart and adding a NOS inhibitor improved the systolic function.

Liver Cirrhosis and Cardiac Dysfunction in CCM: Diastolic Dysfunction

**Figure 2:**
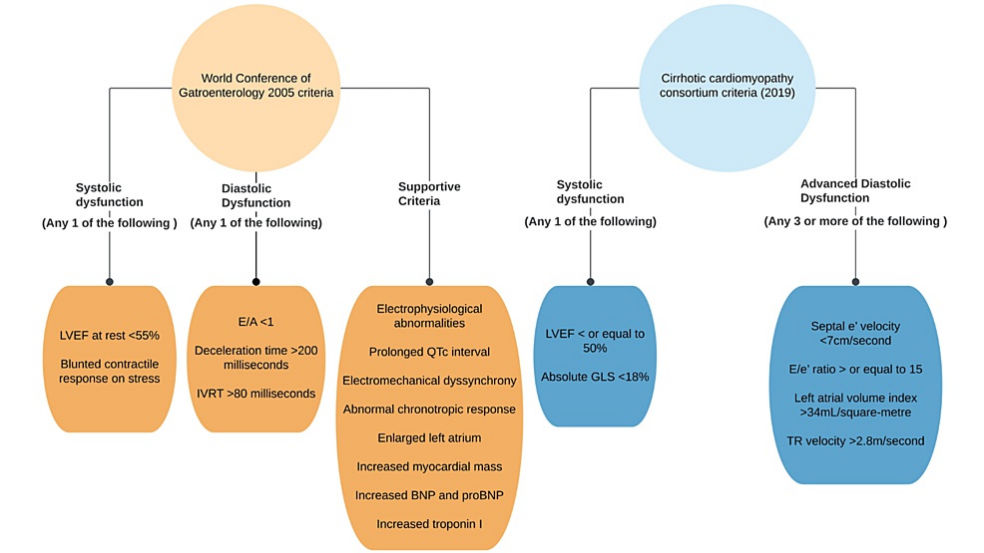
Diagnostic and defining criteria of CCM CCM: Cirrhotic cardiomyopathy, LVEF: Left ventricular ejection fraction, E: Early ventricular filling velocity, A: Late/atrial ventricular filling velocity, IVRT: Isovolumetric relaxation time, QTc: Corrected QT interval, GLS: Global longitudinal strain, e’:  End diastolic mitral annular velocity Image credits: Figure created by author Revanth Kalluru

Diastolic dysfunction generally refers to a ventricular filling defect likely due to a stiffened ventricular wall leading to an elevation of pulmonary venous pressure. Since the contractile function of the myocardium is usually unaffected, EF is preserved [45]. Diastolic dysfunction in CCM, as per the definition given at the 2005 World Conference of Gastroenterology, refers to any one of prolonged deceleration time (DT >200ms), prolonged isovolumetric relaxation time (IVRT >80ms), or the ratio of early (E) to late or atrial (A) phases of LV filling velocities < 1 (E:A ratio <1) (figure 2) [46]. The defining parameters mentioned above are transmitral blood flow parameters measured by a conventional pulsed wave Doppler to assess left ventricular diastolic function [73]. The E wave corresponds to the early phase of ventricular relaxation, where the blood starts filling the ventricular chamber until both the left atrial and the left ventricular pressures equalize. The A wave represents blood added to LV by atrial systole in the late stage of ventricular relaxation. The E-wave deceleration time (DT) represents the time interval between peak E-wave velocity and the end of the E wave [73]. In contrast to systolic dysfunction, which is masked at rest, diastolic dysfunction can be easily revealed using the transmitral blood flow parameters [23]. Abnormal left ventricular relaxation owing to decreased left ventricular compliance leads to the increased IVRT and impedes transmitral blood flow into the LV, prolonging DT. This means that the early left ventricular filling is impaired and leads to the atrial systole making an increased contribution to the left ventricular filling, thereby decreasing the E/A ratio [16].

The fundamental change in diastolic dysfunction is decreased ventricular compliance, and the underlying mechanisms of the pathogenesis are broadly due to persistent activation of vasoconstrictor systems (Figure 1) and alterations in the collagen fibers [12,23,74-76]. Saner et al. disclosed a histologic image of the myocardium from post-mortem biopsies which revealed myocardial hypertrophy, fibrosis, and variations in the size of cells and nuclei shapes [12]. The potent vasoconstrictor systems (RAAS and SNS) play a role in myocardial hypertrophy and fibrosis [16,26]. The SNS and RAAS are believed to act via TGF-β expression, which is identified to be a potent pro-apoptogenic and pro-fibrogenic cytokine in one way or another [26,66,74]. The SNS can induce inflammatory cytokine production, including TGF-β, and induce cardiomyocyte apoptosis and fibrosis [16,66]. The RAAS system is thought to exert its effects in two ways, via direct effects of angiotensin II and salt overload. In a study performed by Kim et al. on rats concluded that angiotensin II (AT II) upregulated extracellular matrix protein synthesis like collagen and TGF-β expression via angiotensin II type 1 (AT 1) receptor [74]. Administration of an AT 1 receptor inhibitor reduced extracellular matrix protein, TGF-β expression, and regressed cardiac hypertrophy [74]. The TGF-β is further believed to act via MAPKs among other pathways, and induce cardiomyocyte apoptosis and fibrosis [66]. Salt loading owing to continuous salt and water retention by RAAS may cause concentric cardiac hypertrophy through activation of cardiac aldosterone independent of RAAS [75]. On a biochemical level, a study performed by Glenn et al. on BDL rats concluded that collagen I increased and a more compliant collagen III decreased in cirrhotic rats [76]. Titin, another passive tension regulator, underwent reduced post-translational modification owing to decreased protein kinase A (PKA) level and may lead to a rise in passive tension in the myocardium. Also, PKA-mediated calcium dissociation from troponin C may reduce, resulting in an increased myocardial relaxation time [76].

Diastolic dysfunction is believed to be more prevalent than systolic dysfunction and may also precede systolic dysfunction as it is present independent of compensated or decompensated cirrhosis which is in contrast to systolic function [16]. Surprisingly insertion of TIPS led to the aggravation of HF bringing diastolic dysfunction into light in liver cirrhosis [17]. A study performed in 1999 by Huonker et al. to understand the effect of TIPS placement in a group of 17 alcoholic cirrhotics concluded that nine hours after TIPS placement, left atrial diameter increased by 6%, left ventricular end-diastolic volume increased by 7%, and no significant change was noticed at the end-systolic volume [77]. Moreover, pulmonary capillary wedge pressure (a reflection of left atrial and left ventricular end-diastolic pressure) increased after nine hours, suggesting that following TIPS placement, splanchnic volume shunting into central circulation led to an increase in preload to the heart. The heart failed to accommodate a sudden spike in preload, resulting in increased back pressure precipitating HF [77]. Large volume paracentesis, however, showed opposite results by improving diastolic dysfunction. The E/A ratio increased, and E-wave deceleration time decreased following paracentesis, implying increased transmittal flow of blood following an increased preload to the heart [30]. This led to a hypothesis that large volume paracentesis led to decompression of the splanchnic vascular bed, allowing further splanchnic vasodilation and a decrease in afterload on the heart [26,30,35].

Liver Cirrhosis and Cardiac Dysfunction in CCM: Electrophysiologic Abnormalities

Electrophysiological changes observed in cirrhotics are a part of the supportive criteria used to diagnose CCM, according to the 2005 World Conference of Gastroenterology [46]. Prolonged QT interval, abnormal chronotropic response, and electromechanical dyssynchrony of the action potential comprise electrophysiologic abnormalities mainly. Prolonged QT is a hallmark and the most common electrophysiologic finding in liver cirrhosis [27]. Prolonged QT interval is reported in half of liver cirrhosis patients and was independent of the etiology of liver disease [5,27]. A study performed in 2003 by Trevisani et al. to assess the role of portal hypertension in QT interval prolongation in a group of 10 patients with non-cirrhotic portal hypertension (NCPH) and 19 cirrhotic patients before and after TIPS placement concluded that QT interval was prolonged in both the groups and QT interval worsened after TIPS placement [78]. These findings suggested that QT prolongation is independent of the etiology of liver disease and a potential role for cardioactive substances shunting into the systemic circulation in the pathogenesis of QT prolongation [78]. The QT interval shows diurnal variation as autonomic tone varies from daytime to nighttime and varies due to several other factors. Thus a corrected QT interval (QTc) is considered more reliable [27]. A constellation of factors may be associated with QT prolongation in CCM, of which cardioactive substances shunting into the systemic circulation, ion channel remodeling, and autonomic dysfunction have been discussed [78].

Cardiac ion channel remodeling, especially the potassium (K^+^) channels, is thought to be one of the primary mechanisms contributing to QT interval prolongation. Animal models of cirrhosis described a decrease in all the three types of K^+^ channel currents which are, Ca^2+^ independent transient outward K^+^ current, delayed rectifying K^+^ current, and the inwardly rectifying background K^+^ current [79]. The inwardly rectifying background K^+^ current is thought to mainly influence the late phase of repolarization and maintain resting membrane potential in the cardiomyocyte. The other two currents play a role initial phase of repolarization [80]. High circulating levels of angiotensin II inhibit K^+^ channels, leading to a more prolonged action potential as repolarization may not immediately follow depolarization, and prolonged repolarization leads to QT interval prolongation [26,79]. A sudden shift to longer action potential is associated with an immediate decrease in peak Ca^2+^ influx current plus a slower decline of the intracellular Ca^2+^ [81]. These together may explain a prolonged contracted state and impaired relaxation of the myocardium [26,79-81]. Plasma membrane fluidity is essential for the normal function of membrane receptors and ion channels. An increase in plasma membrane rigidity due to multifactorial etiology was observed in CCM [82]. This led to a decline in β- adrenergic receptor density, a decline in cAMP production (β-adrenergic receptor-mediated), and K^+^ ion channel alterations [79,82]. Thus altered plasma membrane fluidity is believed to contribute to systolic dysfunction and electrophysiologic abnormalities in CCM [5,79,82]. Autonomic dysfunction (SNS hyperactivity and vagal impairment) also influences QT prolongation. Henriksen et al. showed that plasma noradrenaline levels were associated with prolonged QT intervals, suggesting that SNS influenced QT prolongation in the presence of altered membrane receptors and ion channels [83]. Another study showed that a sudden rise in SNS activity following acute bleeding led to a significant rise in QTc interval [84].

The QT prolongation is well associated with Torsades de Pointes (a polymorphic ventricular tachycardia), which might lead to ventricular fibrillation and sudden cardiac death [85]. However, the incidence of these fatal arrhythmias is rare in the setting of liver cirrhosis [27]. The link between QT interval prolongation and mortality is contradictory, with specific evidence suggesting that QT intervals longer than 440 ms led to a significantly lower survival rate and other evidence suggesting the contrary [86,87]. Administering drugs with QT prolongation action may be avoided or used with dose adjustment and close electrocardiography monitoring [27,88]. Several cases of Torsades de Pointes have been reported following the administration of QT-active drugs as the liver is a significant site for drug metabolism [88]. In contrast to ventricular arrhythmias, more frequently associated arrhythmias with liver cirrhosis are atrial flutter and fibrillation [89]. Liver cirrhosis is hypothesized to be both arrhythmogenic and protective against atrial fibrillation [27].

Electromechanical dyssynchrony refers to an increase in time taken for the mechanical systolic response to follow the electrical depolarization stimulus in the heart [16]. This delay in the electromechanical coupling of an action potential is reported in liver cirrhosis [90]. A study performed in 1991 by Bernardi et al. on a group of 22 patients with liver cirrhosis and 10 controls of similar age to determine the effect of SNS on cardiovascular responsiveness concluded that at rest, cirrhotic patients had higher plasma norepinephrine, prolonged electromechanical delay, and pre-ejection periods compared to controls [90]. Following exercise, an increase in HR and diastolic blood pressure were lesser in cirrhotics. Further, a decrease in pre-ejection time and pre-ejection time to left ventricular ejection time ratio were also lesser in cirrhotics following exercise (Table 2) [90]. These findings further improve our understanding of decreased cardiovascular response to stress.

**Table 2:** A summary of studies to understand diastolic dysfunction and electrophysiologic abnormalities in cirrhosis QTc: corrected QT, TIPS: Transjugular intrahepatic portosystemic shunt, CCM: Cirrhotic cardiomyopathy, BDL: Bile duct ligated, SHRSP rats: Stroke prone spontaneously hypertensive rats,  AT 1: Angiotensin II type 1, TGF-beta: Tumor necrosis factor –beta, ECM: Extracellular matrix

Reference	Population	Methods	Results	Conclusions
Bernardi et al. (1991) [90]	32 patients (22 liver cirrhotics and 10 controls)	Systolic time intervals (electromechanical delay, pre-ejection period, and pre-ejection period to left ventricular ejection time ratios) and plasma noradrenalin levels were monitored before and after exercise.	At rest, cirrhotic patients had higher plasma norepinephrine and prolonged systolic time intervals. After exercise, an increase in heart rate and diastolic blood pressure were lesser in cirrhotics. Further, a decrease in pre-ejection period and pre-ejection time to left ventricular ejection time ratio were also lesser in cirrhotics following exercise.	Inability to increase cardiac performance following sympathetic drive may also be due to defective electromechanical coupling.
Trevisani et al. (2003) [78]	29 patients (10 patients with non-cirrhotic portal hypertension and 19 with cirrhotic portal hypertension)	QTc (corrected QT) interval was compared between the two groups.	Baseline maximum QTc was prolonged (>440 ms) in both groups. Maximum QTc values did not significantly vary between the groups.	QT prolongation is independent of the etiology of liver disease and cardioactive substances shunting into the systemic circulation may cause QT prolongation.
Huonker et al. (1999) [77]	17 alcoholic cirrhotic patients with recent variceal bleeding	Cardiovascular parameters were evaluated before and after TIPS insertion based on echocardiography and catheterization of blood vessels.	Nine hours after TIPS insertion, left atrial diameter increased by 6%, 101% increase in left atrial pressure, 111% increase in pulmonary capillary wedge pressure was noticed.	Insertion of TIPS can unmask the underlying diastolic dysfunction and can precipitate signs and symptoms of heart failure in CCM patients.
Glenn et al. (2011) [76]	BDL rats and control sham-operated rats	Cardiomyocyte proteins titin and collagen were measured by Western blot analysis, and diastolic function and passive tension of the ventricular wall were examined.	Titin mRNA underwent reduced post-translational modification. Stiffer collagen type I increased and a more compliant collagen type III reduced.	Altered titin and collagen configuration lead to Impaired relaxation of the myocardium and a rise in passive tension of the ventricular wall.
Kim et al. (1995) [74]	SHRSP rats and control Wistar Kyoto rats.	Northern blot analysis of AT 1 antagonist mediated gene expression of TGF-beta and ECM proteins.	SHRSP rats had an increased gene expression for TGF- beta and ECM proteins. Following AT1 receptor antagonist administration, the expression of those genes reduced significantly.	Administration of an AT 1 receptor antagonist reduced extracellular matrix protein, TGF-β expression, and regressed cardiac hypertrophy.

Diagnosis

During the 2005 World Conference of Gastroenterology, an entity called CCM was defined, and diagnostic criteria were proposed (Figure 2) [46]. Echocardiographic parameters based on tissue pulse wave Doppler were proposed to diagnose cardiac dysfunction [46,74]. However, with advancing knowledge, these parameters were found to have limitations, and newer diagnostic modalities emerged over the years. Thus experts from multiple disciplines came together to update the diagnostic criteria from CCM, forming the Cirrhotic Cardiomyopathy Consortium criteria in 2019 [46].

The LVEF could be more than 55% at rest in CCM due to the hyperdynamic circulation [5,26]. Regarding the contractile response to stress, cirrhotic patients are often put on β adrenergic receptor blockers. The LVEF reflects only a part of the contractile function of the myocardium [46,91]. Cardiac strain refers to the pattern of deformation the myocardium undergoes during systole, and this deformation is quantified by echocardiographic strain imaging or myocardial deformation imaging. It is more sensitive than conventional echocardiography [92,92]. Left ventricular strain consists of longitudinal, radial, and circumferential strain. Radial strain represents the thickening of the left ventricular wall during systole. Circumferential strain refers to the contraction of the myocardial outline in the anteroposterior and lateral-medial axis during systole. Longitudinal strain is the contraction of the myocardium along the base to the apex of the heart direction during systole. The LVEF mainly represents the radial strain on the heart during systole [92]. Global longitudinal strain (GLS) is believed to be affected earlier than radial function [93]. Strain values are described in percentages as they denote a change in length during systole compared to a baseline length of the diastole. However, GLS is a negative value as the myocardium shortens in this direction during systole; absolute values are preferred to avoid confusion [46,92]. The CCM Consortium criteria recommend absolute GLS <18% in cirrhotic patients with a normal LVEF (>50%) to be diagnostic of systolic dysfunction (as seen above in Figure 2) [46].

The diastolic dysfunction parameters proposed in the 2005 diagnostic criteria are all preload-dependent (Figure 2) [73]. For example, prolonged LV relaxation/increasing preload may slow down the early (E) phase of left ventricular filling leading to a decrease in E velocity. However, it may eventually elevate the left atrial pressure, increasing the E velocity [94]. Similarly, E deceleration time and IVRT are both affected by the progression of diastolic dysfunction and by an alteration of preload conditions [73]. These parameters pose two problems: (1) an underlying progression of the disease process or altering preload can influence these parameters, and (2) since, with the progression of the disease these parameters oppose each other, they may show pseudo-normal values in the later stages of the disease masking the condition [73]. Thus, patients with normal and advanced diastolic dysfunction may show similar values [46]. Alternatively, early diastolic mitral annular velocity (e') is less preload-dependent [46]. Due to the longitudinal strain of the myocardium in a cardiac cycle, the mitral valve annulus moves toward the apex during systole and away from the relatively stationary apex in diastole. The e' denotes mitral annular velocity in early diastole and reflects myocardial relaxation [46]. Since diastolic dysfunction is thought to precede systolic dysfunction and is characterized by impaired relaxation, medial or septal e' and E/e' ratios have been proposed as its markers [16,46]. Left atrial volume is a reflector of diastolic dysfunction and denotes chronic elevation of left ventricular filling pressures [95]. Further tricuspid regurgitation (TR) velocity can be a marker for pulmonary hypertension. According to the CCM Consortium criteria, advanced diastolic dysfunction suggested by 2016 modified American Society of Echocardiography (ASE) guidelines is considered diagnostic of diastolic dysfunction in CCM in the absence of prior heart disease (Figure 2) [46].

Treatment

Cirrhotic cardiomyopathy is often asymptomatic and goes undiagnosed until it presents later with signs of HF in the acute decompensated stage of cirrhosis or after TIPS placement [30]. Further, a specific pharmacological therapy strictly targeting CCM does not exist yet, and liver transplantation remains the way to cure CCM [16]. However, attempts are being made to target specific pathophysiologic areas of CCM. Treatment is supportive in nature, meaning it is initiated only where HF becomes apparent and is nonspecific, focusing on general principles of HF treatment in the absence of cirrhosis [5]. These are mainly fluid and salt restrictions and promote their excretion using diuretics [96]. Administration of positive ionotropic agents to improve contractility are ineffective due to the chronotropic incompetence in CCM [48]. Reduction in the afterload further in the presence of underlying arterial hypotension may have adverse effects [16]. Thus leaving diuretics as the mainstay of treatment [16,23,96].

Aldosterone antagonists like spironolactone are indicated apart from fluid and salt excretion, as they also inhibit myocardial fibrosis and activation of SNS, which are considered effects of aldosterone [96]. A study performed in 2005 by Pozzi M et al. to determine the effect of long-term treatment with aldosterone antagonists in a group of 22 cirrhotic patients and 10 age-matched controls concluded that aldosterone antagonist (K-canreonoate) significantly reduced left ventricular wall thickness, LVEDV, and hepatic venous pressure gradient. However, they noticed no improvement in the E/A ratio and proposed an additive effect of β blockers and aldosterone antagonists to play a role in improving cardiac dysfunction in CCM patients [96]. Angiotensin-converting enzyme inhibitors are contraindicated as they might exacerbate the systemic vasodilation in an already vasodilated vascular bed [5]. Although they promoted sodium excretion, angiotensin II receptor blockers did not show long-term beneficial effects [97].

Nonselective β-blockers are a mainstay in treating portal hypertension and preventing variceal bleeds [91]. They also improve QT interval prolongation and electromechanical dyssynchrony [98]. However, their administration decreased CO and increased mortality rates in patients with refractory ascites [99]. Further, whether improving the QT interval impacts the incidence of life-threatening arrhythmias and mortality benefit is yet to be discovered [16,27]. Insertion of TIPS to treat variceal hemorrhage leads to shifting blood into the central circulation, increasing preload on the heart. Diastolic dysfunction is preload sensitive, and a sudden shift in circulation may precipitate signs and symptoms of heart failure. However, these findings normalize around six months following the procedure [17].

Liver transplantation acutely corrects metabolic dysfunction and increases the SVR. This led to a drop in LVEF due to an underlying cardiac dysfunction further exacerbated by an increase in afterload on the heart and only unmasked the underlying systolic dysfunction [49]. However, LVEF completely recovered following liver transplant in a follow-up study [49]. Liver transplantation is the only effective treatment that can improve systolic and diastolic dysfunction. The QT prolongation was reversed in about half of the patients following a transplant [27,100]. A study performed by Terregrosa et al. in 2005 on a group of 40 cirrhotics and 15 controls to assess reversibility of cardiac changes in CCM post liver transplant concluded that liver transplant normalized impaired systolic response to exercise, regressed ventricular wall thickness, and diastolic dysfunction [100]. Liver transplantation, however, poses a significant risk of perioperative and postoperative complications like heart failure, myocardial infarction, and arrhythmias. A complete cardiac evaluation is recommended prior to transplantation for all liver transplantation candidates [27].

It is evident that a lack of well established-diagnostic guidelines and limitations in the treatment options demands a compelling need to develop new and specific agents to treat CCM. A new agent, 2 (acetyloxy) benzoic acid-3 (nitrooxymethyl) phenyl ester (NCX-1000), aimed at releasing the vasodilator NO inside the liver and intrahepatic production of a vasodilator hydrogen sulfide via farnesoid X receptor, is currently of pharmacologic interest. Other potential areas are CB1 receptor antagonism, NOS inhibitor, and TGF-β blockers [16,101,102].

Limitations

This review provides an overall picture of the various mediators involved in the pathogenesis of CCM. Describing in depth molecular mechanism of each mediator is beyond the scope of this article.

Cardiac dysfunction in liver cirrhosis is also believed to drive the pathophysiology of hepatopulmonary syndrome. This review solely focuses on the relationship between the liver and the heart and briefly mentions their joint role in the development of HRS. Protective and pro-arrhythmogenic influence on atrial fibrillation in the presence of liver cirrhosis has been mentioned. However, an in-depth understanding and the outcomes in this setting are not delved into completely.

## Conclusions

Many liver cirrhosis patients have an underlying, often underdiagnosed, cardiac dysfunction. The disease process may be present even in the compensated phase of liver cirrhosis, especially diastolic dysfunction. Although present from the initial stages of cirrhosis, a normal or high CO and LVEF at rest render the patient asymptomatic. This masking poses a direct challenge to early diagnosis and prognostic outcomes of the condition. Cardiac dysfunction plays a crucial role in the development of HRS. Insertion of TIPS and liver transplantation may unmask an underlying cardiac dysfunction leading to signs of heart failure, which may worsen the intervention outcomes. This syndrome often remains undiagnosed due to a lack of well-established diagnostic criteria and limitations of transmitral flow parameters estimated by simple echocardiography. Through this review article, we have attempted to convey the clinical significance of an underlying cardiac dysfunction in patients with liver cirrhosis. The GLS value by myocardial strain imaging enables clinicians to pick up systolic dysfunction even at rest when LVEF is normal. Utilizing e' and E/e' ratios in the diagnostic criteria allows a more accurate diagnosis of impaired myocardial relaxation. A complete cardiac evaluation before a procedural intervention and close monitoring for the development of cardiac decompensation is recommended to prevent post-procedural complications like heart failure. The QT interval-prolonging drugs are ideally avoided or dose adjusted with close electrocardiography monitoring. Liver transplantation remains the only effective treatment that can correct cardiac dysfunction.

This article aims to provide a comprehensive understanding of the mechanisms leading up to cardiac dysfunction and discusses newer parameters proposed by the Cirrhotic Cardiomyopathy Consortium to diagnose CCM. We believe a lack of specific pharmacological therapy demands further understanding of the disease pathophysiology and clinical trials in this area. Further, developing more sensitive diagnostic markers and strict diagnostic criteria may significantly improve the prognosis of the patients.
